# Hierarchical Population Genetic Structure in a Direct Developing Antarctic Marine Invertebrate

**DOI:** 10.1371/journal.pone.0063954

**Published:** 2013-05-14

**Authors:** Joseph I. Hoffman, Andrew Clarke, Melody S. Clark, Lloyd S. Peck

**Affiliations:** 1 Department of Animal Behaviour, University of Bielefeld, Bielefeld, Germany; 2 British Antarctic Survey, Natural Environment Research Council, High Cross, Cambridge, United Kingdom; University of Bologna, Italy

## Abstract

Understanding the relationship between life-history variation and population structure in marine invertebrates is not straightforward. This is particularly true of polar species due to the difficulty of obtaining samples and a paucity of genomic resources from which to develop nuclear genetic markers. Such knowledge, however, is essential for understanding how different taxa may respond to climate change in the most rapidly warming regions of the planet. We therefore used over two hundred polymorphic Amplified Fragment Length Polymorphisms (AFLPs) to explore population connectivity at three hierachical spatial scales in the direct developing Antarctic topshell *Margarella antarctica*. To previously published data from five populations spanning a 1500 km transect along the length of the Western Antarctic Peninsula, we added new AFLP data for four populations separated by up to 6 km within Ryder Bay, Adelaide Island. Overall, we found a nonlinear isolation-by-distance pattern, suggestive of weaker population structure within Ryder Bay than is present over larger spatial scales. Nevertheless, significantly positive *F*_st_ values were obtained in all but two of ten pairwise population comparisons within the bay following Bonferroni correction for multiple tests. This is in contrast to a previous study of the broadcast spawner *Nacella concinna* that found no significant genetic differences among several of the same sites. By implication, the topshell's direct-developing lifestyle may constrain its ability to disperse even over relatively small geographic scales.

## Introduction

Marine species exhibit extreme heterogeneity in dispersal ability as estimated from genetic data [1] but despite decades of study, the underlying factors are not yet fully understood. One factor that has received a great deal of attention is life-history, since contrasting strategies can either facilitate or hinder dispersal, leading to predictions about the extent to which different species are likely to exhibit population structure [1]–[3]. Specifically, direct developers with restricted dispersal capabilities are hypothesised to be more structured than otherwise ecologically equivalent species with pelagic larvae. In addition, pelagic larval duration is expected to correlate negatively with genetic differentiation.

Although several recent meta-analytical and comparative studies lend support to these theoretical expectations [1]–[9], others have not been able to establish clear links between life-history traits and the strength of population structure [10]–[18]. Such discrepancies could result from any of a large number of potentially confounding factors including larval or adult behaviour [14], [19], the degree of ecological specialisation [11], [20], differences in effective population size [20] or sweepstakes-like reproductive strategies [15]. Moreover, technical factors such as the choice of genetic marker [16], sample size [3] and the completeness of geographic sampling [21] may also play an important role.

In addition to the above factors, our ability to draw general conclusions in respect of the relationship between life history and population structure is also hindered by a bias in the literature towards species from low latitudes [2]. To obtain a more representative view of global patterns therefore requires a broadening of focus to include under-represented geographical and ecological regions such as the poles. Polar species are of particular interest because their development rates are often around 5–10 times slower than equivalent temperate species [22], [23], leading to exceptionally long larval durations in broadcast reproducers. Furthermore the effects of uniquely polar ecological factors such as ice disturbance on population structure have been little studied [24] despite their pervasive and frequent impact on shallow sites [25].

Antarctica provides an unparalleled opportunity to undertake studies of the origins and maintenance of biological diversity, both at the levels of species and populations [26]. Millions of years of isolation from warmer waters to the North by the Antarctic Circumpolar Current have led to the evolution of a diverse and abundant benthic fauna that is both highly endemic and cold adapted [27], [28]. However, parts of Antarctica are now experiencing anthropogenically induced warming at a rate that may soon outstrip the ability of many species to adapt physiologically [29], [30]. For example, sea temperatures have increased by 1°C to the west of the Antarctic Peninsula in the last 50 years [31] and in parallel mean annual air temperatures on the Antarctic Peninsula have increased by as much as 3°C over the same period [32]. This has driven the widespread retreat of glaciers, ice shelf collapse and the exposure of new habitats in both terrestrial and marine locations. In turn, extensive areas of new intense biological productivity have been generated together with associated nearshore benthic ecosystems [33]. This places a premium on genetic studies capable of documenting ‘baseline’ patterns of population structure, which in turn may help to predict the capacity of species or populations to adapt to environmental change [34].

Consistent with predictions based on life history, several Antarctic brooding species have been found to be so highly structured as to invoke cryptic speciation [35]–[39]. However, other brooding species appear unexpectedly homogenous or at least show evidence of gene flow over relatively large geographic scales [40], [41]. Moreover, contradictory findings have also been obtained for several different Antarctic broadcast spawning species [42]–[45]. This suggests the need for comparative studies that are able to control for as many incidental factors as possible, for instance by sampling co-distributed species from the same geographic localities, collecting equal sample sizes of individuals and using the same class of genetic marker.

**Figure 1:**
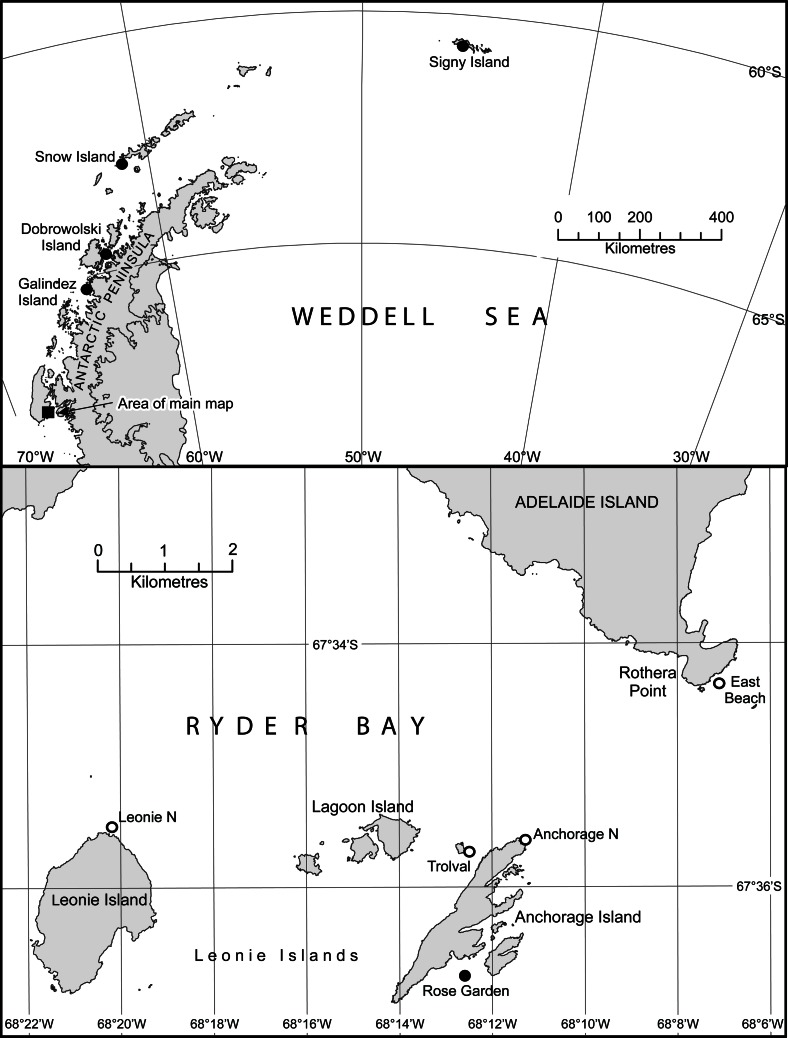
Map showing *M.* *antarctica* sampling locations. Populations sampled and analysed as part of the current study are denoted by white points, while those sampled previously by Hoffman *et al.* [46] are denoted by black points. The upper panel shows the Antarctic Peninsula and the lower panel shows the Ryder Bay area (Adelaide Island). Figure modified from [46].

To evaluate the role of life-history variation within an Antarctic setting, Hoffman *et al*. [46] recently used Amplified Fragment Length Polymorphisms (AFLPs) to analyse paired populations of the broadcast-spawning Antarctic limpet *Nacella concinna* and the brooding topshell *Margarella antarctica* sampled at five locations along the Antarctic Peninsula (see Figure 1 for the sampling sites). The broadcast-spawner was found to be panmictic across most of the peninsula, with only two populations from the extremes of the range, Adelaide and Signy Islands, being genetically differentiated. In contrast, population structure was seven times stronger overall in *M.antarctica*, with model-based clustering approaches assigning all of the individuals correctly to their source populations based solely on their multilocus genotypes.

An intriguing aspect of the above study was the finding that *N.concinna* populations from Adelaide Island, at the base of the Antarctic Peninsula, were significantly differentiated from the other Antarctic peninsula populations despite being connected by continuous coastline and this species possessing long-lived planktotrophic veliger larvae [47]. To explore this further, the original AFLP study was extended to include additional *N. concinna* populations from three localities within Ryder Bay, Adelaide Island (Rothera Point, Leonie and Anchorage Islands), each of which was in turn sub-sampled three times to provide a fine-scale geographic perspective [48]. Surprisingly, limpets sampled from Rothera Point and Leonie Island could not be distinguished on the basis of their AFLP profiles from populations sampled further afield along the Antarctic Peninsula, whereas all the three sub-populations from Anchorage Island showed slight but significant genetic differences. One potential explanation for this is that coastal eddies around Anchorage Island could be advecting larvae back towards the shore, a mechanism invoked to explain local ‘hot spots’ of larval retention encountered in computer simulations [49]. Alternatively, the exposed location of Anchorage Island on the outermost edge of Ryder Bay could predispose it to occasional sporadic larval input from sites to the south [50]. Another possibility relates to the fact that, due to the uneven retreat of coastal glaciers and ice shelves, habitats present at Anchorage Island may be thousands of years older than those around Rothera Point [51]. Consequently, limpet populations across Ryder Bay could have experienced markedly different demographic histories.

The above studies raise additional questions in a comparative context. For example, are *M. antarctica* populations within Ryder Bay more structured than those of *N.concinna* as predicted by differences in their life history? Also, does life history influence the pattern of microgeographic population structure, or is this shaped in both species by the same underlying processes? To address these questions, we expanded our previous macrogeographic *M. antarctica* dataset to incorporate 174 additional individuals sampled from four sites over a 1–6 km spatial scale within Ryder Bay. All 414 individuals were genotyped at 228 polymorphic AFLP loci.

## Materials and Methods

### Tissue sample collection

*M. antarctica* samples were collected by SCUBA divers during the austral summer of 1999 from the shallow sublittoral zone off Rothera Point (East Beach), Leonie Island (Leonie North North East) and from Anchorage North and Trolval on Anchorage Island (see Table 1 and Figure 1 for details). Samples were stored in 95% ethanol, initially for four months at −20°C and thereafter at room temperature. Previously published AFLP data from Rose Garden (Anchorage Island), Galindez, Dobrowolski, Snow and Signy Islands [46] were also available for comparison. The combined dataset allowed us to investigate population structure over three spatial scales as follows: (i) Fine-scale (up to 2 km, within Anchorage Island) comprising samples from Rose Garden, Anchorage North and Trolval; (ii) Medium-scale (up to 6 km, among islands within Ryder Bay) comprising samples from Anchorage Island, Rothera Point and Leonie Island; (iii) Large scale (up to 1350 km, along the Antarctic Peninsula) comprising samples from Ryder Bay, Galindez Island, Dobrolowski Island, Snow Island and Signy Island. Geographic distances among these localities are given in Table 2.

**Table 1:** Details of sampling locations and numbers of *M. antarctica* individuals collected, including 240 individuals previously sampled by Hoffman *et al.* [46].

Region	Site	Population	Latitude (S)	Longitude (W)	No. of samples
Ryder Bay	Anchorage Island	Rose Garden	−67.607	−68.191	48
		Anchorage North	−67.602	−68.202	44
		Trolval	−67.608	−68.218	43
	Rothera Point	East Beach	−67.572	−68.118	43
	Leonie Island	Leonie North	−67.603	−68.336	44
Galindez Island	–	–	−65.233	−64.233	48
Dobrolowski Island	–	–	−64.917	−62.607	48
Snow Island	–	–	−62.778	−61.374	48
Signy Island	–	–	−60.677	−45.607	48
Total	–	–	–	–	414

The three spatial scales investigated were as follows (i) Fine-scale (within Anchorage Island) comprising samples from Rose Garden, Anchorage North and Trolval; (ii) Medium-scale (among islands within Ryder Bay) comprising samples from Anchorage Island, Rothera Point and Leonie Island; (iii) Large scale (along the Antarctic Peninsula) comprising samples from Ryder Bay, Galindez Island, Dobrolowski Island, Snow Island and Signy Island.

**Table 2:** Matrix of geographic distances among the nine *M. antarctica* sampling sites in kilometres.

	Rose Garden	Anchorage North	Trolval	Rothera Point	Leonie Island	Galindez Island	Dobrowolski Island	Snow Island	Signy Island
Rose Garden	0								
Anchorage North	0.66	0							
Trolval	0.71	0.64	0						
Rothera Point	2.98	2.50	3.12	0					
Leonie Island	3.62	3.56	2.99	5.70	0				
Galindez Island	304.18	303.66	304.22	301.20	305.88	0			
Dobrowolski Island	370.17	369.59	370.09	367.25	371.28	88.27	0		
Snow Island	635.07	634.53	635.07	632.10	636.57	331.84	270.97	0	
Signy Island	1342.37	1342.10	1342.74	1339.71	1345.37	1083.96	1076.25	866.43	0

### DNA extraction and AFLP genotyping

Total genomic DNA was extracted from a small piece of foot tissue from each individual using a Qiagen DNeasy tissue extraction kit following the manufacturer's recommended protocols. We then used an AFLP protocol adapted from Vos et al. [52] as detailed by Hoffman et al. [53] that employed seven different selective primer combinations (Table 3). PCR products were resolved by electrophoresis through 6% polyacrylamide gels and exposed to X-ray film for five days. These were developed using a universal X-ray developer (Xograph Healthcare Ltd.) and, if required, a second exposure was made for an adjusted time period. All bands in the approximate size range of 75–300 bp were scored manually by an experienced operator (JIH). Only clear bands with minimal size variation were included, these being recorded as 1 = present and 0 = absent. Pairs of bands that were clearly non-independent were scored as single traits. It was assumed that AFLP bands that were the same size across individuals represented homologous markers. In combining our new data with those previously published, we also revisited the original autoradiographs and were able to score a small number of additional loci across all of the samples.

**Table 3:** Primer combinations used for the AFLP selective amplification and numbers of AFLP bands generated in 414 *M. antarctica* individuals.

*Taq*I primer (5′-3′)	*Eco*RI primer (5′-3′)	Number of loci	Number of polymorphic loci	% of polymorphic loci
GATGAGTCCTGACCGA–CTG	GACTGCGTACCAATTC–AGC	47	42	89.4
GATGAGTCCTGACCGA–CGA	GACTGCGTACCAATTC–AGC	42	37	86.1
GATGAGTCCTGACCGA–CAG	GACTGCGTACCAATTC–AGC	30	22	73.3
GATGAGTCCTGACCGA–CAC	GACTGCGTACCAATTC–AGC	32	25	78.1
GATGAGTCCTGACCGA–CCA	GACTGCGTACCAATTC–AAC	29	20	69.0
GATGAGTCCTGACCGA–CGA	GACTGCGTACCAATTC–ATG	44	38	86.4
GATGAGTCCTGACCGA–CAG	GACTGCGTACCAATTC–ATG	52	44	84.6
Total		276	228	82.6

### Data analysis

The program Aflp-Surv [54] was used to calculate global *F*_st_ together with pairwise *F*_st_ values among all of the populations. Statistical significance was determined using permutation tests based on 10,000 randomisations of the dataset. Geographic distances among populations were calculated using a Geographic Information System (ESRI ArcGis v 9.2) as described in detail by Hoffman et al. [45]. The significance of correlations between genetic and geographic distance was assessed using Mantel tests with 999 iterations implemented in Genalex v6 [55].

## Results

### Overall population structure

**Figure 2:**
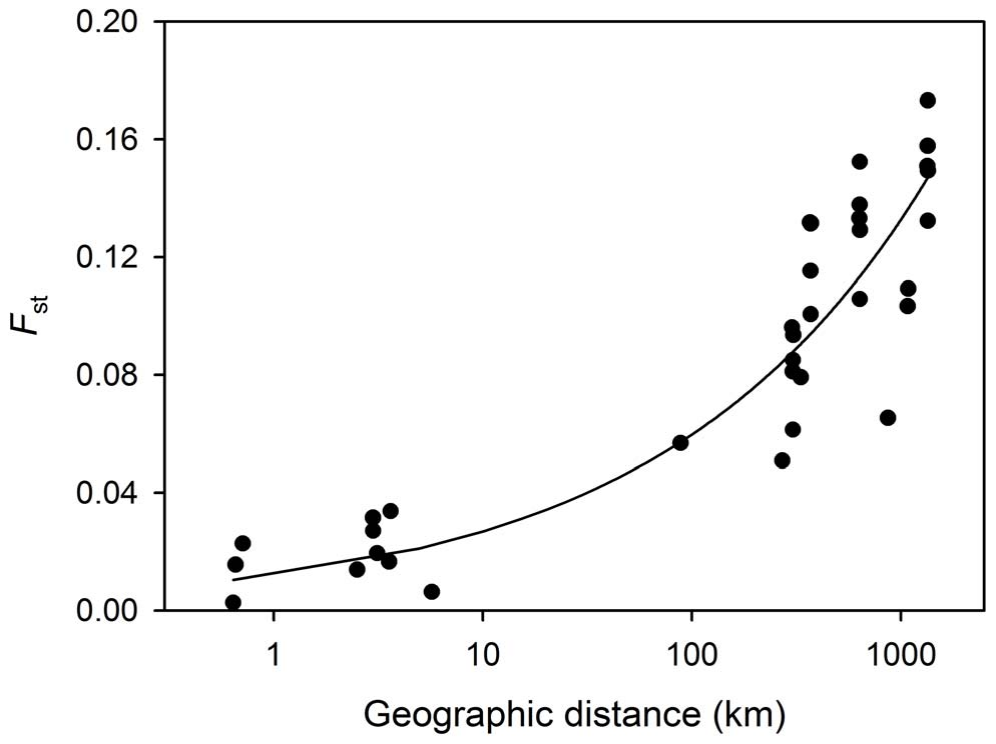
The relationship between geographic and genetic distance (*F*st) among nine *M.* *antarctica* populations. To indicate the underlying trend, a power regression was fitted (y = 0.0121×^0.3467^, *r* ^2^ = 0.811), which explains a marginally greater proportion of the variance than logarithmic or second order polynomial regressions (*r* ^2^ = 0.777 and 0.782 respectively).

To a previous dataset comprising five *Margarella antarctica* populations spanning the Antarctic Peninsula [46] we added new AFLP data for four populations within Ryder Bay, Adelaide Island (See Table 1 and Figure 1 for details). The resulting dataset comprised 414 individuals scored for 276 AFLP bands, of which 228 (82.6%) were polymorphic (Table 3). A permutation test for genetic differentiation among all nine populations based on 10, 000 randomisations of the dataset indicated a strong deviation from the null hypothesis of no genetic structure (*F*_st_ = 0.0872, *P*<0.0001). Restricting the analysis to the five populations sampled within Ryder Bay, global *F*_st_ was lower (0.0181) but still highly significant (*P*<0.0001), indicating the presence of fine-scale population genetic structuring. The relationship between geographic and genetic distance was highly significant overall (Figure 2, Mantel's *r* = 0.00, *n* = 9, *P*<0.001), with a power regression fitting the data considerably better (*r* ^2^ = 0.811) than linear, logarithmic or second order polynomial regressions (*r* ^2^ = 0.641, 0.777 and 0.782 respectively). Restricting the analysis to populations within Ryder Bay, the isolation-by-distance relationship remained positive but failed to reach statistical significance (Mantel's *r* = 0.09, *n* = 5, *P* = 0.383).

### Population structure within Ryder Bay

*F*_st_ values for each of the pairwise population comparisons within Ryder Bay ranged from 0.003 to 0.034 (Table 4). All but one of these values were individually significant, six out of ten being so at *P*<0.0001. The only comparison failing to reach significance was that between Anchorage North and Trolval, the two geographically closest populations sampled from a single Island, Anchorage. Following table-wide Bonferroni correction for multiple statistical tests, *F*_st_ between Leonie Island and Rothera Point also became no longer significant (*P* = 0.076).

**Table 4:** Pairwise *F*_st_ values among *M. antarctica* sampled from nine different sites (above diagonal).

	Rose Garden	Anchorage North	Trolval	Rothera Point	Leonie Island	Galindez Island	Dobrowolski Island	Snow Island	Signy Island
Rose Garden	*	0.016	0.023	0.032	0.034	0.061	0.101	0.106	0.132
Anchorage North	0.0002	*	0.003	0.014	0.017	0.081	0.115	0.138	0.158
Trolval	<0.0001	0.0578	*	0.020	0.027	0.085	0.131	0.152	0.173
Rothera Point	<0.0001	0.0005	<0.0001		0.006	0.096	0.132	0.133	0.151
Leonie Island	<0.0001	<0.0001	<0.0001	0.0022	*	0.094	0.132	0.129	0.149
Galindez Island	<0.0001	<0.0001	<0.0001	<0.0001	<0.0001	*	0.057	0.079	0.109
Dobrowolski Island	<0.0001	<0.0001	<0.0001	<0.0001	<0.0001	<0.0001	*	0.051	0.103
Snow Island	<0.0001	<0.0001	<0.0001	<0.0001	<0.0001	<0.0001	<0.0001	*	0.065
Signy Island	<0.0001	<0.0001	<0.0001	<0.0001	<0.0001	<0.0001	<0.0001	<0.0001	*

*P*-values, calculated using 10,000 permutations of the dataset, are given below the diagonal. All of the *F*_st_ values were significant at *P*<0.05, except for that between Anchorage North and Trolval. After table-wide Bonferroni correction for multiple statistical tests, *F*_st_ the comparison between Rothera Point and Leonie Island also became non-significant.

## Discussion

Although studies of the population genetic structure of marine species are commonplace, relatively few have explicitly compared direct with indirect-developers, particularly over multiple geographic scales and using the same methodologies with samples from the same sites. Consequently, we extended previous work on the brooding top shell *M. antarctica* to allow a comparison over three hierarchical spatial scales with the broadcast-spawning limpet *N. concinna*. Consistent with expectations, *M. antarctica* populations were structured throughout Ryder Bay, with the overall magnitude of genetic differentiation being greater than previously found in equivalent comparisons involving *N. concinna*. This lends further support to the notion that life history could be an important determinant of population structure in many benthic marine species.

### Strength and patterns of population genetic structure

Because the previous study of *M. antarctica* sampled only five populations from the Western Antarctic Peninsula [46], this did not allow inferences to be drawn in respect of geographic scales below 100 km. Specifically, it was unclear whether the isolation-by-distance relationship could be linear, which would imply strong microgeographic population structure, or whether this could instead break down over finer geographic scales. By adding data from four populations within Ryder Bay, we were able to resolve a non-linear profile, with a power regression providing a greatly improved fit to the data relative to a linear one (*r* ^2^ = 0.811 versus 0.661, Figure 2). By implication, population structure appears to be far weaker over microgeographic than macrogeographic scales. This is consistent with a recent study of a brooding sea urchin that found genetic differences between patches separated by around ten metres, but little in the way of genetic structure within patches [34]. In contrast, however, distances of several kilometres appear to be just as effective a barrier to dispersal as far larger regions of unsuitable habitat in a direct-developing cushion star [7]. The reasons underlying such interspecific differences remain unclear, partly due to a paucity of empirical data. These may be specific to the species studied or could instead result from generic regional effects such as markedly stronger seasonality and temporally restricted resources, ice scour in polar regions, or the slowed development and delayed maturity characteristic of low temperature species.

Despite population structure being relatively weak within Ryder Bay, global *F*_st_ was still significantly non-zero (0.0181, *P*<0.0001) and around twenty times larger than the equivalent value for *N. concinna* based on the same five populations (0.0009, *P = *0.024). Such a marked difference could potentially reflect the influence of Antarctic conditions on the two species' life histories. For example, larval lifetimes tend to be longer in species adapted to cold climates [22], [56], [57], thereby extending the duration of the dispersal phase and hence the scope for gene flow. This leads to the prediction that, if all other factors could be controlled for, differences in population structure between brooders and pelagic developers could be greater at high latitudes than low latitudes. However, set against this, polar marine species tend to have disproportionately long lifespans (e.g. *Laternula elliptica* can live for up to 36 years [58] and *Adamussium colbecki* for over 100 years [59]). This could conceivably allow more time for adults of direct-developing gastropod species to migrate from site to site where suitable substratum and depth allow.

A related observation is that *M. antarctica* and *N. concinna* not only differed in the strength of population structure, but also in the way in which genetic variation was partitioned within Ryder Bay. All but two pairwise comparisons in *M. antarctica* were statistically significant following Bonferroni correction for multiple tests, suggesting that population structure although weak may extend across much if not all of the bay. In contrast, most of the *N. concinna* populations from Ryder Bay were indistinguishable from one another, with only three populations from Anchorage Island revealing significant genetic differences [48]. These contrasting patterns suggest that different factors may be influencing local population structure in the two species. One possibility is that coastal eddies or sporadic larval input from outside the bay could be disproportionately important in the broadcast spawner. To allow generalisation beyond the single comparison we have drawn here, it would be interesting to sample additional brooding and broadcasting species from across the bay, the expectation being that broad similarities should be found within, but not between the two classes of organism.

Another factor that could potentially contribute to population structure in *M. antarctica* is benthic topology. Although Ryder Bay is only 15–20 km across, it attains a maximum depth of over 500 metres [60], which is beyond the distribution depth of many shallow-water marine species. There is also considerable variation in benthic topology and substratum. The latter could be important because the capacity of *M. antarctica* to disperse over soft sediments is greatly diminished relative to hard substrata such as bed rock or loose rubble. This hypothesis would be amenable to testing using a ‘landscape genetics’ approach if sufficiently fine-scale data could be collected on depth and substrate distribution across Ryder Bay. It is noteworthy in this context that the only comparison in which we did not obtain a significant *F*_st_ value prior to Bonferroni correction involved the two geographically closest *M. antarctica* populations, Trolval and Anchorage North, which were situated less than one kilometre apart on the coast of Anchorage Island. These two sites are separated by rocky coast and around 100 m of soft sediment at depths of 30–40 m (L. Peck pers. obs). However both sites are on the north side of Anchorage Island where steep to vertical rocky slopes descend to beyond 300 m. It is therefore possible that gene transfer between these two sites could take place along the rocky continuum beyond 40 m.

### Caveats and future directions

This study was made feasible through the use of AFLPs because these markers are capable of generating large numbers of genome-wide distributed bands in virtually any organism, including diverse Antarctic marine taxa [61], with little need for optimisation. Although we were able to score a large number of bands, however, the dominant nature of these markers leads to increased variance in the estimation of allele frequencies [54]. Furthermore, AFLP bands are typically assumed to be independent whereas in reality mutations, insertions or deletions can generate bands of different size that are linked [62]. This is difficult to control for experimentally, although we attempted to do so by scoring only bands that were clearly independent from one another. Similarly, not all bands of the same size are likely to be homologous [63], [64], although manual scoring may help to alleviate this problem because size homoplasious bands representing different loci may show varying intensities as well as some conformation or sequence dependent differences in electrophoretic mobility [65]. Many of the above factors could be eliminated in future studies by deploying Single Nucleotide Polymorphisms (SNPs), since these markers are codominant and can be mapped to a reference genome to ensure selection of an unlinked, genome-wide distributed panel. Moreover, fully automated genotyping and scoring methods allow SNPs to be genotyped with minimal error [66].

As with any type of genetic marker, AFLPs can also be difficult to compare across species boundaries because different subsets of bands will be generated and these will invariably differ both in number and polymorphic information content. However, we believe this is unlikely to account for the contrasting strength and patterns of genetic structure observed in this study, since the number of informative bands obtained for *M. antarctica* and *N. concinna* individuals from Ryder Bay was fairly similar at 189 and 155 respectively, and the smaller panel is still reasonably large. Classical analyses of population structure also assume that all loci are selectively neutral, but some studies have reported conflicting results for different types of marker, suggesting that this may not always be the case [67]. This is implicit in the widespread use of AFLPs for conducting genome scans for ‘outlier loci’ that may be influenced by natural selection [68], [69], although this approach is rapidly being superceded by Restriction Site Associated DNA (RAD) sequencing and allied approaches that draw upon emerging high-throughput sequencing technologies [70]. These could potentially be employed in the future to determine whether outlier loci could be important in the context of this particular study.

Elsewhere, several factors other than life-history have also been invoked to explain the population structure of marine species. For example, Galarza et al. [14] found no relationship between either egg type or larval duration and the strength of genetic structure among seven littoral fish species, leading these authors to suggest that genetic connectivity could be influenced by larval or adult behaviour. Similarly, evidence from two comparative studies [13], [71] suggests that habitat specificity may also have a strong impact upon population structure. Another influential factor could be effective population size [20] since genetic drift occurs more quickly in small populations, suggesting that highly abundant species could be relatively predisposed towards being unstructured. Finally, local extinctions and recolonisations or extreme heterogeneity in the reproductive success of individuals may also lead to temporal fluctuations in population structure [72], [73]. To account for these and other factors presents a major challenge, since this would require both temporal sampling and the inclusion of additional phylogenetically diverse species.

Finally, although our study design allowed us to control for technical factors such as the sampling scheme, choice of genetic marker and the genotyping and scoring protocols, we were not able to include further replicates at the same spatial scale from elsewhere that would help disassociate our findings from any conditions that could be specific to Ryder Bay. Detailed lichenographic studies suggest that the retreat of ice has been uneven across the bay, leaving behind a patchwork of habitats of varying ages [51], so we cannot exclude the possibility that the two species recolonised at different rates or from different sources, or that they could have responded differently to demographic challenges such as bottlenecks induced by the ebb and flow of sea ice. This criticism could be partly addressed by incorporating multi-level sampling from other geographical regions. However, Ryder Bay was an ideal choice for this particular study due to its proximity to the British Antarctic Survey research base at Rothera Point together with detailed knowledge of the local area.

## Conclusion

This study extended previous work on the direct developing top shell *M. antarctica* to include a microgeographic component. Overall, a strong but nonlinear isolation-by-distance pattern was detected, indicating relatively weak population structure over scales of 1–6 km. Nevertheless, this was consistent across the bay and greater in magnitude than previously documented for the broadcast spawner *N. concinna*. By implication, life history variation, specifically protected versus broadcast reproductive modes, may impact the population structure of benthic marine species over multiple geographic scales. This could have implications for understanding how Antarctic marine invertebrates may respond to climate change, since dispersal is a key means by which locally extirpated populations might be replenished from adjacent locations.
